# Biomarkers for Diagnosing Febrile Illness in Immunocompromised Children: A Systematic Review of the Literature

**DOI:** 10.3389/fped.2022.828569

**Published:** 2022-03-10

**Authors:** Fabian J. S. van der Velden, Andrew R. Gennery, Marieke Emonts

**Affiliations:** ^1^Translational and Clinical Research Institute, Newcastle University, Newcastle upon Tyne, United Kingdom; ^2^Great North Children's Hospital, Paediatric Immunology, Infectious Diseases and Allergy, Newcastle upon Tyne Hospitals NHS Foundation Trust, Newcastle upon Tyne, United Kingdom

**Keywords:** pediatric, fever, biomarkers, immunocompromised, diagnostics

## Abstract

**Objective:**

This study aims to assess the performance of biomarkers used for the prediction of bacterial, viral, and fungal infection in immunocompromised children upon presentation with fever.

**Methods:**

We performed a literature search using PubMed and MEDLINE and In-Process & Other Non-indexed Citations databases. Cohort and case–control studies assessing biomarkers for the prediction of bacterial, viral, or fungal infection in immunocompromised children vs. conventional microbiological investigations were eligible. Studies including adult patients were eligible if pediatric data were separately assessable. Data on definitions used for infections, fever, and neutropenia and predictive values were collected. Risk of bias was assessed with the Quality Assessment of Diagnostic Accuracy Studies-2 tool.

**Results:**

Fifty-two studies involving 13,939 febrile episodes in 7,059 children were included. In total, 92.2% were in cancer patients (*n* = 48), and 15.7% also included hematopoietic stem cell transplantation patients (*n* = 8). Forty-three biomarkers were investigated, of which 6 (CRP, PCT, IL-8, IL-6, IL-10, and TNFα) were significantly associated with bacterial infection at admission, studied in multiple studies, and provided predictive data. Literature on the prediction of viral and fungal infection was too limited. Eight studies compared C-reactive protein (CRP) and procalcitonin (PCT), with PCT demonstrating superiority in 5. IL-6, IL-8, and IL-10 were compared with CRP in six, four, and one study, respectively, with mixed results on diagnostic superiority. No clear superior biomarker comparing PCT vs. IL-6, IL-8, or IL-10 was identified.

**Discussion:**

There is great heterogeneity in the biomarkers studied and cutoff values and definitions used, thus complicating the analysis. Literature for immunocompromised children with non-malignant disease and for non-bacterial infection is sparse. Literature on novel diagnostics was not available. We illustrated the challenges of diagnosing fever adequately in this study population and the need for improved biomarkers and clinical decision-making tools.

## Introduction

Febrile illness is common in children, accounting for 20% of emergency department (ED) attendances (1). Often a self-limiting (viral) illness, 10–15% of febrile children in ED will have serious bacterial infection (SBI) or sepsis (1–4).

Diagnosing fever is challenging in immunocompromised children; often fever is the only symptom. They are at a high risk (HR) for SBI as their immune system cannot combat pathogens adequately. Up to half of solid tumor and ≥80% of hematological malignancy patients have ≥1 episode of febrile neutropenia (5). Moreover, 11.4–31.3% of febrile neutropenic children have positive blood cultures, and 41.3–62.3% have no cause identified (6–9).

Common biomarkers, such as C-reactive protein (CRP) and procalcitonin (PCT), aid us but are often not sensitive enough to rule out a bacterial infection or differentiate other causes of fever (10–12), resulting in admissions for virtually all febrile immunocompromised children for intravenous antibiotics pending microbiological results. The introduction of this approach led to a significant reduction in mortality and morbidity (13).

It is suspected that a significant proportion of febrile illness is deemed viral in nature, caused by medication or the underlying disease itself (14). Unnecessary admissions and antibiotic use could be avoided and the quality of life for patients and families could be improved (15) if diagnostic tests could rule out bacterial infection in ED.

There is a need to investigate new diagnostic markers and clinical decision-making tools to improve the diagnosis of febrile illness in this group. Externally validated tools often exclude the immunocompromised (16). Transcriptomic techniques are increasingly investigated for distinguishing bacterial and viral causes of fever in ED but are not clinically validated (17–19). In immunocompromised patients, two promising studies (20, 21) used transcriptomics to identify biomarkers for bacteremia in patients with febrile neutropenia, but both included adults.

This literature review assessed the biomarkers for diagnosing the etiology of febrile illness in immunocompromised children and their performance.

## Methods

The research question for this review was as follows: “Which biomarkers have been studied for diagnosing the etiology of febrile illness in immunocompromised children, and how well do they perform?”

We searched PubMed and MEDLINE and In-Process and Other Non-indexed Citations databases and used the PRISMA 2020 reporting guidelines (22) for this review.

The PubMed search was carried out using the following terms: [“Infant, Newborn”(MeSH) OR “Infant”(MeSH) OR “Child”(MeSH) OR “Child, Preschool”(MeSH) OR “Adolescent”(MeSH)]

AND [“Neoplasms”(MeSH) OR “Immunologic Deficiency Syndromes”(MeSH) OR “Neutropenia”(MeSH) OR “Immunocompromised Host”(MeSH) OR “Hematopoietic Stem Cell Transplantation”(MeSH) OR “Transplant Recipient”(MeSH) OR “Fever”(MeSH) OR “Febrile Neutropenia”(MeSH) OR “neutropenic fever”(TIAB)]

AND [“Biomarkers”(MeSH) OR “Cytokines”(MeSH)] AND [“Bacterial Infections”(MeSH) OR “Virus Diseases”(MeSH) OR “Mycoses”(MeSH) OR “Bacteremia”(MeSH) OR “Sepsis”(MeSH)]. The MEDLINE search is described in Supplementary Data 1.

No language restrictions nor any filters were applied, and relevant Spanish or German publications were translated.

Based on the Population, Intervention, Comparison, Outcome, and Settings (22) criteria, the inclusion criteria were HR children ≤ 18 years of age presenting with fever or suspected infection. HR children were immunocompromised due to primary (PID) or secondary immunodeficiency, with biomarkers assessed for the predictive ability of bacterial, viral, or other infection. The outcomes considered were as follows: SBI, bacteremia, viremia, fungemia, clinically documented infection, microbiologically documented infection, sepsis, septic shock, clinical deterioration, and pediatric intensive care unit (PICU) or high dependency unit admission. These had to be cohort or case–control studies. Studies combining children and adults were considered if pediatric data was assessable separately.

### Data Extraction and Bias Assessment

Study data were collected on demographics, the study's inclusion and exclusion criteria, definitions used, clinical characteristics, biomarkers, and outcome.

For every biomarker, data were extracted on cutoff values, specificity, sensitivity, and positive and negative predictive values. If unavailable, outcome assessment was performed by using reported measures of central tendency plus spread.

Risk of bias was assessed using the Quality Assessment of Diagnostic Accuracy Studies (QUADAS-2) tool (23), which was slightly modified: the quality item “time between index test and reference test were appropriate” was not deemed discriminative because the index (biomarker) and the reference test (conventional microbiology) were obtained simultaneously.

## Results

**Figure 1:**
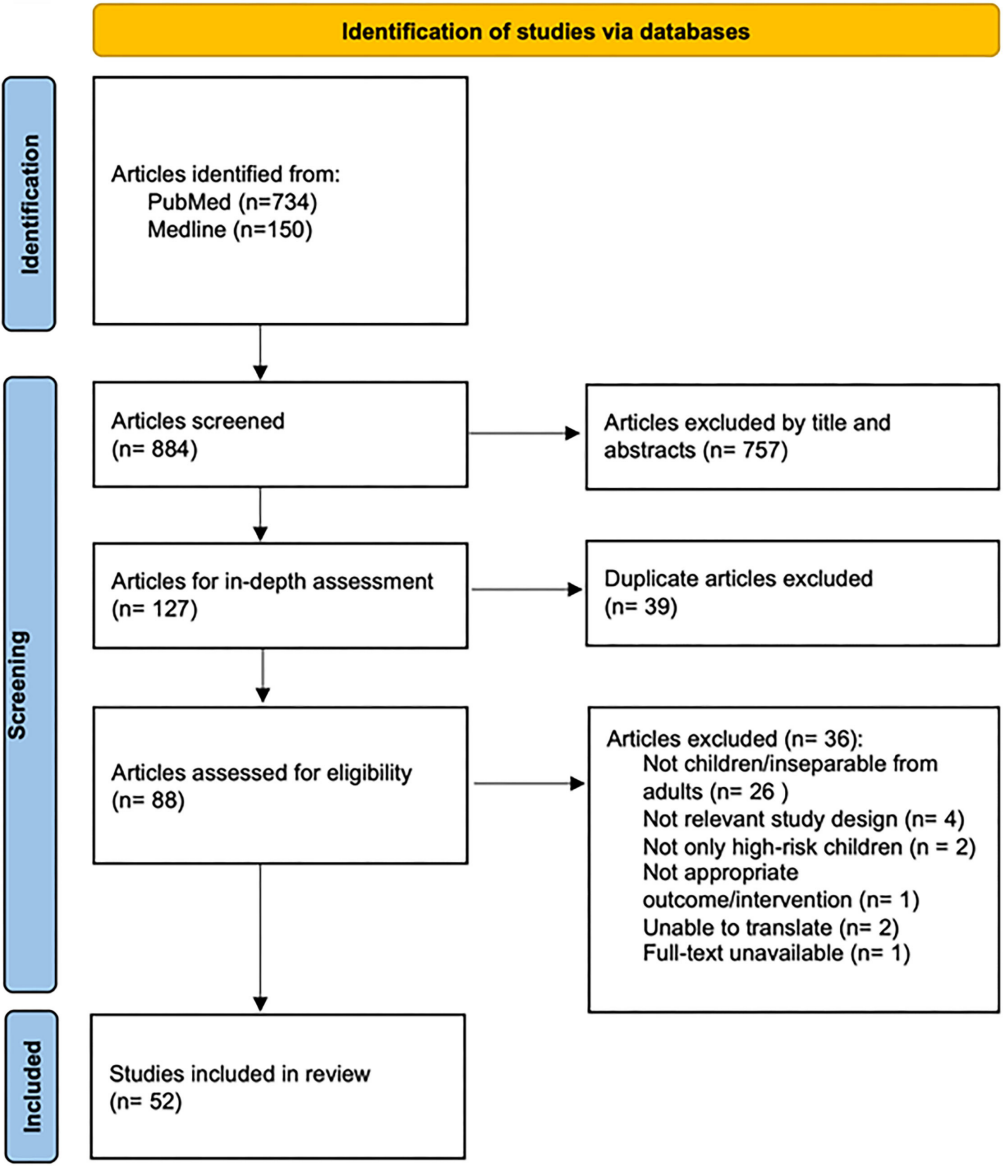
Flow chart of study identification and inclusion, adopted from PRISMA 2020 guidelines.

Database searches were performed on November 23, 2021 and yielded 884 potential articles. Eighty-eight studies were analyzed in-depth after title and abstract screening and removal of duplicates. Thirty-six studies were excluded: pediatric data inseparable from adult data (*n* = 26), inclusion of non-HR patients (*n* = 2), not relevant study design (*n* = 4), not in English or unable to translate (*n* = 2), inappropriate interventions or outcomes (*n* = 1), and full text unavailable (*n* = 1). In total, 52 articles were included (Figure 1).

### Study and Population Characteristics

Across the included studies, 13,939 episodes of 7,059 children from 20 countries were analyzed. The median age ranged between 3.0 and 9.9 years. Eight studies did not provide age data for their whole cohort, only subgroups (24–31).

The majority of studies used pediatric cancer patients as their immunocompromised group (92.2%), and 8 (15.7%) explicitly stated the inclusion of hematopoietic stem cell transplant (HSCT) patients. Three studies (5.9%) included solid organ transplant recipients (Table 1).

**Table 1:** Characteristics of the included studies.

**Citation**	**Median age, years (IQR, years)**	**High-risk condition (*n*)**	**Biomarkers studied [timepoint (s)]**	**Number of patients**	**Number of episodes**	**Endpoints studied**	**Comments on endpoints**	**Fever definition**	**Neutropenia definition**
Aquino et al. (32)	Mean 7.6 ± 3.3	Malignancy (excludes HSCT)	CRP, ESR, exotoxin, IFNγ, IL-1β, IL-2, IL-4, IL-5, IL-6, IL-8, IL-10, TNFα, MIP-1α, MIP-1α, MCP-1, protein C (admission)	47	58	Bacteremia, LOS		T ≥38.5°C or T ≥38.0°C ≥2 times over 4-h interval	ANC <0.5 × 10^9^/L
Ashkenazi-Hoffnung et al. (33)	8.4 (2–17)	Renal or renal + liver transplant	CRP (admission)	52	168	CDI, MDI, ICU admission, mortality (in-hospital)	Bacterial vs. other causes of fever	T ≥38.0°C	Not stated
Badurdeen et al. (24)	Not stated	Malignancy	CRP, IL-1, IL-5, IL-6, IL-8, IL-10, IL-12p70 (unclear timepoint)	44	55	Bacteremia		T >38.5°C or sustained T >38.0°C	ANC <1.0 × 10^9^/L
Baraka and Zakaria (9)	Mean 8.72 ± 3.55	hematological malignancy	CRP, PCT, presepsin (unclear timepoint)	90	90	CDI, MDI	Group Ia: FUO, group Ib bacteremia; group Ic clinically proven infection; healthy matched-control group	T ≥38.3°C or T ≥38.0°C sustained over 1-h interval	ANC 0.5 × 10^9^/L or ANC dropping to 0.5 × 10^9^/L in 24–48 h
Cabanillas Stanchi et al. (34)	8.5 (range 0.4–17.8)	Allogenic HSCT (day 0 until discharge)	CRP, PCT (admission)	214	214	Infection/sepsis, GvHD	Bacteremia, viremia, fungemia, mucositis, other causes of fever	Not stated	ANC <0.5 × 10^9^/L
Delebarre et al. (35)	Mean 7.6 ± 5.1	Malignancy	CRP, PCT (unclear timepoint)	160	372	Severe infection	Severe infection: bacteremia, positive culture from sterile site, invasive fungal infection, or local infection with risk of extension	T ≥ 38.5°C or T ≥ 38.0°C ≥2 times over 6-h interval	ANC <0.5 × 10^9^/L
Diepold et al. (36)	7.7 (range 0.8–20)	Malignancy, HSCT	CRP, IL-6, IL-8 (admission, day 2, day 3)	69	141	Infection, sepsis, FUO, prolonged fever	Group: FUO + ≤ 3 days of fever, group: FUO + ≥5 days of fever (includes clinical sepsis and localized infection), group: sepsis (only blood culture positive)	T ≥ 38.3°C or T ≥ 38.0°C sustained over 1-h interval	ANC <0.5 × 10^9^/L
Doerflinger et al. (37)	6.0 (3.4–11.3)	hematological malignancy, solid malignancy	CRP, IL-6, IL-8, MIP-1α, PCT (admission, day 2)	64	79	Bacteremia, CDI, MDI, FUO	Bacteremia vs. rest	T ≥ 38.0°C	ANC < 1.0 × 10^9^/L
Döring et al. (38)	7.0 (range 0.5–26)	HSCT	IL-1β, sIL-2R, IL-6, IL-8, IL-10, TNFα (pre-HSCT baseline; onset of symptoms)	61	61	Sepsis, bacteremia, fungemia, viremia, GvHD	Compared with individual's baseline	Not stated	Not stated
El-Maghraby et al. (39)	Mean 7.1 (range 1.5–18)	Haematological malignancy	CRP, IL-8, MCP-1 (admission)	76	85	Bacteremia, sepsis, localized infection, FUO		T > 38.5°C once or T >38.0°C sustained over 6-h interval	ANC < 0.5 × 10^9^/L
Gunasekaran et al. (25)	Not stated	Malignancy, HSCT	PCT (admission)	316	821	MDI, CDI, mortality	Secondary analysis of bacterial, viral, and fungal infection	T > 38.3°C or T > 38.0°C ≥2 times over 1-h interval	ANC < 1.0 × 10^9^/L
Hatzistilianou et al. (40)	Mean 5.9 ± 2.9	ALL	CRP, IL-6, PCT, neoptrin, NO_2_/NO_3_ (admission; daily until day 7)	37	208	Bacteremia, clinical bacterial infection, viral infection, FUO, drug-induced fever	Also compared to afebrile neutropenic and afebrile non-neutropenic matched controls	T ≥38.5°C or T ≥ 38.0°C ≥2 times over 6-h interval	ANC < 0.5 × 10^9^/L or, if ANC unknown, WBC <1.0 × 10^9^/L
Hatzistilianou et al. (41)	Mean 5.8 ± 2.9	Acute leukemia	CRP, IL-1β, IL-8, PCT, sTNF-R2 (admission, serial daily until day 7)	104	221	CDI, MDI, FUO	Group A: bacterial infection, neutropenic; group B: viral infection and FUO; group C bacterial, not neutropenic; group D: viral and FUO, not neutropenic, group E: no fever, not neutropenic	T > 38.5°C once or T > 38.0°C sustained over 6 h	ANC < 0.5 × 10^9^/L or, if ANC unknown, WBC < 1.0 × 10^9^/L
Hazan et al. (42)	Mean 9.5 ± 5.9	Malignancy	CRP, WBC (admission, daily until discharge)	73	195	Bacteremia	Blood culture at admission positive or new blood culture pathogen ≥10 days after bacteremic episode, followed by the documented disappearance of the first pathogen	T ≥ 38.3°C or T ≥ 38.0°C sustained over a minimum 1-h period	ANC <0.5 × 10^9^/L
Heney et al. (43)	Mean 7.0 (range 0.6–15.0)	Malignancy	CRP, IL-6 (admission, daily until discharge)	33	47	Gram-negative bacteremia, gram-positive bacteremia, FUO	Groups defined by blood culture yield	T > 38.5°C once or T > 38.0°C twice <24 h	Not stated
Hodge et al. (26)	Not stated	Malignancy	CRP, IL-5, IL-8, IL-12 (admission)	31	31	MDI	Blood culture positive *vs*. blood culture negative	T > 38.5°C or sustained T >38.0°C	ANC <1.0 × 10^9^/L
Hodge et al. (27)	Not stated	Malignancy	IL-2, IFNγ, TNFα, Tregs (CD25+, CD127−, CD8–, CD3+) (admission)	26	27	Bacteremia	Blood culture positive *vs*. blood culture negative	T > 38.5°C or sustained T > 38.0°C	ANC < 1.0 × 10^9^/L
Jacobs et al. (44)	7.8 (3.1–13.8)	HSCT (any neutropenic timepoint), solid organ transplant, malignancy	IL-27, PCT (admission)	293	400	MDI, CDI, mortality (in hospital, 28-day, 60-day)	Bacterial infection *vs*. other causes of fever	Not stated	ANC < 0.5 × 10^9^/L
Jacobs et al. (45)	7.8 (3.1–13.8)	HSCT (any neutropenic timepoint), solid organ transplant, malignancy	GZMB, HSPA1B, IL-8, CCL3, MMP-8 (admission)	293	400	Clinical deterioration <72 h, MDI, 28-day mortality	Severity biomarkers in suspected bacterial infection	Not stated	ANC < 0.5 × 10^9^/L
Karakurt et al. (46)	Mean 8.0 (range 0.3–17)	Non-leukemia malignancy	CRP, IL-6, IL-8, sIL-2R, PCT, sTNF-R2 (admission)	31	50	CDI, MDI, LOS, mortality	Severity biomarkers in suspected bacterial infection	T > 38.5°C once or T ≥ 38.0°C ≥2 times in 12 h	ANC < 0.5 × 10^9^/L or ANC 0.5–1.0 × 10^9^/L expected to fall <0.5 × 10^9^/L ≤ 72 h
Kassam et al. (47)	6.2 (3.9–13.8)	Malignancy	Protein C (<48 h of admission)	73	73	Bacteremia, CDI		T ≥ 38.3°C or T ≥ 38.0°C sustained over 12 h	ANC < 0.5 × 10^9^/L
Kitanovski et al. (48)	7.6 (range 1.0–18)	Malignancy	CRP, IL-6, PCT (day 1, day 2, day 3)	32	68	Bacteremia/clinical sepsis, localized infection, FUO	Localized infection defined by clinical features or focal culture positive	Not stated	ANC < 0.5 × 10^9^/L or ANC 0.5–1.0 × 10^9^/L expected to fall <0.5 × 10^9^/L ≤ 24–48 h
Kitanovski et al. (6)	6.3 (range 0.5–19.9)	Malignancy	CRP, LBP, PCT, IL-6 (admission, 24 h)	48	90	Bacteremia/sepsis, local infection, FUO, other causes	Bacteremia/sepsis: blood culture positive or clinically documented sepsis; other causes: fungemia, viral and paraneoplastic fever	T ≥ 38.5°C or T ≥ 38.0°C ≥2 times <12 h	ANC < 0.5 × 10^9^/L or ANC 0.5–1.0 × 10^9^/L expected to fall <0.5 × 10^9^/L ≤ 48 h
Lehrnbecher et al. (49)	Mean 8.0 (range 0.3–20.0)	Malignancy	CRP, IL-6, IL-8, sFc??RIII, MBP (admission; 24 h)	56	121	MDI, CDI, FUO	Gram-negative bacteremia *vs*. other causes of fever	T >38.5°C or T ≥ 38.0°C ≥2 times over a 4-h interval	ANC <0.5 × 10^9^/L
Lehrnbecher et al. (50)	Mean 9.0 (range 0.5–28.0)	Malignancy	IL-6, IL-8 (admission, 24 h)	146	311	Bacteremia, pneumonia, localized infection, FUO		T > 38.5°C	ANC < 0.5 × 10^9^/L
Li et al. (51)	Mean 3.0 ± 2.1	Solid malignancy	CRP, LDH, PCT (admission)	152	152	Infection, tumor progression	Infection defined as per CDC guidelines (52)	T > 38.0°C	Not stated
Martinez-Albarran et al. (53)	Mean 7.1 ± 5.0	Malignancy	CRP, PCT (admission)	54	54	Low risk vs. high-risk, mortality, complications	Low risk: culture-negative and no clinical evidence of sepsis; high risk: clinical sepsis or blood culture or urine culture-positive sepsis	T > 38.5°C at least ≥2 over a 1-h interval	ANC < 0.5 × 10^9^/L
Miedema et al. (28)	Not stated for whole cohort, only subgroups	Malignancy	CRP, IL-8, PCT, sTREM-1 (admission; 24–48 h)	29	43	Bacterial infection/clinical sepsis group, non-bacterial/clinical sepsis group	Bacterial infection defined by pathogen grown from blood or sterile site culture, radiologically confirmed infection, or clinical sepsis	T≥ 38.5°C or T ≥38.0°C ≥2 times <6 h	ANC <0.5 × 10^9^/L or, if ANC unknown, WBC < 1.0 × 10^9^/L
Miedema et al. (54)	Only range 0.0–18.0	HSCT	CRP, IL-8 (day 1, day 2)	32	52	Bacteremia		Not stated	Not stated
Nath et al. (55)	Mean 6.5 ± 4.1	Malignancy	CRP, PCT (admission)	300	300	Bacteremia		Not stated	Not stated
Özdemir et al. (56)	Mean 8.6 ± 0.83	Malignancy	CRP, PCT, presepsin, sTREM-1 (<24 h of admission; 24–48 h into admission; day 7)	30	47	Bacteremia		T > 38.0°C once or >37.5°C sustained for a minimum of 1 h	ANC < 0.5 × 10^9^/L or ANC 0.5–1.0 × 10^9^/L expected to fall <0.5 × 10^9^/L ≤ 24–48 h
Pacheco-Rosas et al. (57)	8.5 (4.5–11.5)	Malignancy	Lactate (admission)	100	100	Severe sepsis group, non-severe sepsis group	Sepsis definitions as per international guidelines (58)	T ≥38.3°C or T ≥38.0°C sustained over a 1-h period	ANC < 0.5 × 10^9^/L or ANC 0.5–1.0 × 10^9^/L expected to fall <0.5 × 10^9^/L ≤ 24–48 h
Reyna-Figueroa et al. (59)	Mean 9.8 (range 2.0–17.0)	ALL	TNFα, TNF-R1, Fas, Fas-L (admission)	72	72	Bacterial sepsis, organ dysfunction, mortality	Bacterial sepsis: blood culture positive only, not clinical or sterile site	T >38.0°C or T >37.5–37.9°C ≥2 times <24 h	ANC <1.0 × 10^9^/L
Riikonen et al. (60)	Only range 1.0–16.0	Malignancy	CRP, IL-1β, IL-6, SAA, TNFα (admission, day 1, day 2)	46	81	Sepsis/bacteremia, FUO, localized infection, other causes	Group A: blood culture positive; group B: blood culture negative; group C: blood culture negative, focal culture positive; group D: viral and drug-induced	T >39.0°C or T >38.0°C twice in 4-h interval	Severe: ANC <0.2 × 10^9^/L, Moderate: ANC 0.2–0.5 × 10^9^/L, Mild: ANC 0.5–1.0 × 10^9^/L
Riikonen et al. (61)	Only range 1.0–16.0	Malignancy	CRP (admission, 48 h)	Not stated	91	Sepsis/bacteremia, FUO, localized infection, other causes	Group A: blood culture positive; group B: blood culture negative; group C: blood culture neg, focal culture positive; group D viral and drug-induced	T >39.0°C or T >38.0°C twice in 4-h interval	ANC <0.5 × 10^9^/L
Ruggiero et al. (29)	Not stated	Malignancy	CRP, calprotectin, PCT, presepsin (admission, day 3, day 5)	25	39	Bacteremia/clinical sepsis		T ≥38.0°C	ANC <0.5 × 10^9^/L
Sahbudak et al. (62)	7.7 (range 1.1–18)	Malignancy	CRP, IL-6, IL-8, IL-10 (admission, 72 h afebrile post-treatment)	38	59	Bacteremia, Gram-negative bacteremia, fungemia		T ≥38.3°C or T ≥38.0°C sustained over a 1-h period	ANC < 0.5 × 10^9^/L or ANC 0.5–1.0 × 10^9^/L expected to fall <0.5 × 10^9^/L ≤ 48 h
Santolaya et al. (63)	Mean 9.9 ± 5.0	Malignancy	CRP, IL-8, PCT (admission, 24 h)	278	566	Severe sepsis	Only septic children	Not stated	ANC <0.5 × 10^9^/L
Santolaya et al. (64)	Mean 9.2 (no range)	Malignancy (excludes HSCT)	CRP, IL-8 (admission, 24 h)	403	447	Severe sepsis	Sepsis definition as per international guidelines (58)	T ≥38.3°C or ≥38.0°C sustained over a 1-h period	ANC <0.5 × 10^9^/L
Schmidt et al. (65)	Mean 9 (range 0.3–18)	Allogenic HSCT (day 0 to +30)	CRP, PCT (admission, 24 h)	33	33	Bacteremia, FUO		T ≥38.3°C	ANC <0.5 × 10^9^/L
Secmeer et al. (66)	7.7 (range 2.0–18.0)	Malignancy	CRP, ESR, PCT (admission, 8 h, 24 h, 48 h)	49	60	CDI, MDI, FUO	Group I: blood culture positive or clinically defined; group II: non-documented infection	T ≥38.3°C or ≥38.0°C sustained over a minimum 1-h period	Not stated
Soker et al. (67)	7.0 (range 2.0–14.0)	Malignancy	IL-1β, sIL-2R, IL-6, IL-8, TNFα (admission)	23	48	Bacteremia, FUO		T ≥38.5°C or ≥38.0°C twice ≤ 4 h	ANC <0.5 × 10^9^/L
Spasova et al. (68)	Only range 0.2–19.0	Malignancy	CRP (admission, day 3, day 5), IL-6, IL-8, IL10 (admission, 24 h, 72 h)	24	41	Bacteremia, CDI, MDI, focal infection		T ≥38.3°C or T >38.0°C ≥2 times with 1-h interval	ANC <0.5 × 10^9^/L
Stryjewski et al. (13)	6.7 (range 0.4–17.1)	Malignancy	IL-6, IL-8, PCT (admission, 24 h, 48 h)	56	56	Sepsis, septic shock	Sepsis: blood or sterile site culture positive; septic shock as per the modified criteria (69)	T ≥38.0°C	ANC <0.5 × 10^9^/L
Urbonas et al. [IL-10] (70)	Mean 3.0 (range 1.0–17.0)	Malignancy	IL-10 (admission)	24	36	Bacteremia/clinical sepsis, FUO		T ≥38.5°C or T ≥38.0°C twice <6 h	ANC <0.5 × 10^9^/L
Urbonas et al. [IL-6/8] (8)	Mean 7.0 (range 1.0–18.0)	Malignancy	IL-6, IL-8 (admission, 18–24 h)	37	61	Bacteremia/clinical sepsis, FUO		T >38.5°C or T >38.0°C sustained over 6 h	ANC <0.5 × 10^9^/L
Urbonas et al. (71)	6.0 (range 1.0–17.0)	Malignancy	Presepsin, sHLA-G, PCT, sIL-2R (admission)	37	62	Bacteremia/clinical sepsis, FUO		T ≥38.5°C	ANC <0.5 × 10^9^/L
van der Galiën et al. (72)	6.3 (0.8–18.8)	Malignancy	CRP, IL-6 IL-8, PCT (admission, 12–24 h)	55	77	Bacterial infection, no bacterial infection	Bacterial: blood sterile site culture positive or radiologically documented infection	T >38.5°C once or >38.0°C sustained for minimum 6 h	ANC <0.5 × 10^9^/L or, if ANC unknown, WBC <1.0 × 10^9^/L
Vyles et al. (30)	Not stated	ALL	CRP, ESR, PCT (admission)	492	735	Bacteremia		T ≥38.3°C or T ≥38.0°C sustained over a minimum 1-h period	Not stated
Xia et al. (31)	Not stated for cohort, only subgroups	hematological malignancy	CRP, PCT, IL-2, IL-4, IL-6, IL-8, IL-10, TNFα, IFNγ (admission)	992	3,023	CDI, MDI, septicemia, localized infection	Blood culture positive group, blood culture negative infection group, matched-control group; secondary split between bacterial, viral, and fungal	T >38.5°C once or T = 38.0–38.4°C ≥2 times <24 h	ANC <0.5 × 10^9^/L
Xu et al. (73)	8.6 (range 1.2–15.1)	hematological malignancy	IL-2, IL-4, IL-6, IL-10, IFNγ, TNFα (admission)	94	100	Septic shock	All participants had microbiologically documented septic shock; definition by international guidelines (58)	T >38.5°C	ANC <0.5 × 10^9^/L
Xu et al. (74)	6.1 (range 0.1–17.5)	Malignancy	CRP, IFNγ, IL-6, IL-10, PCT, TNFα (admission)	1,115	3,118	Bacteremia, FUO, sepsis severity	Bacteremia and non-bacteremia groups, septic shock and non-septic shock groups, gram-negative bacteremia group	T ≥38.5°C or T ≥38.0°C ≥2 times in <24 h	ANC <0.5 × 10^9^/L

*ANC, absolute neutrophil count; CCL3, C-C chemokine ligand 3; CDI, clinically documented infection; ESR, erythrocyte sedimentation rate; Fas-L, Fas ligand; FUO, fever of unknown origin; GZMB, granzyme B; HSPA1B, heat shock protein 70 kDa 1B; IFNγ, interferon gamma; LBP, lipopolysaccharide-binding protein; LDH, lactate dehydrogenase; MBP, mannose-binding protein; MCP-1, monocyte chemotactic protein-1; MDI, microbiologically documented infection; MIP, macrophage inflammatory protein; MMP-8, matrix metalloproteinase 8; NO_2_, nitrogen dioxide; NO_3_, nitrate; SAA, serum amyloid A; sFcy RIII, soluble Fc gamma receptor type III; SHLA-G, soluble human leucocyte antigen-G; sIL-2R, soluble interleukin 2 receptor; sTNF-R2, soluble tumor necrosis factor receptor-2; sTREM-1, soluble triggering receptor expressed on myeloid cells-1; TNFα, tumor necrosis factor alpha; Tregs, T-regulatory lymphocytes; WBC, white blood cell count*.

The definitions of fever were comparable between the studies, with most using a single temperature ≥38.5°C (40.4%) or ≥38.3°C (21.2%) combined with a sustained temperature ≥38.0°C within 1-, 4-, 6-, 12-, or 24-h interval (59.6%). Eight studies did not formally state their definition of fever.

The definitions of neutropenia were mostly consistent, defined as absolute neutrophil count (ANC) <0.5 × 10^9^/L (*n* = 37, 71.2%), but with 7 studies defining neutropenia as ANC <1.0 × 10^9^/L. Seven studies expanded their definition to include initial ANC <1.0 × 10^9^/L if it is expected to drop to <0.5 × 10^9^/L within 24–72 h. Four studies additionally defined neutropenia as white cell count <1.0 × 10^9^/L if no ANC was available. Eight studies did not state their definitions.

The outcome measures were reported as bacteremia, clinically documented infection, microbiologically documented infection, clinical sepsis, fever of unknown origin (FUO), fungemia, localized infection, sepsis, septic shock, severe infection, and viremia. Other outcomes such as graft-vs.-host disease, length of stay, PICU admission, and prolonged fever were usually not reported in the context of biomarker performance in the included studies.

**Figure 2:**
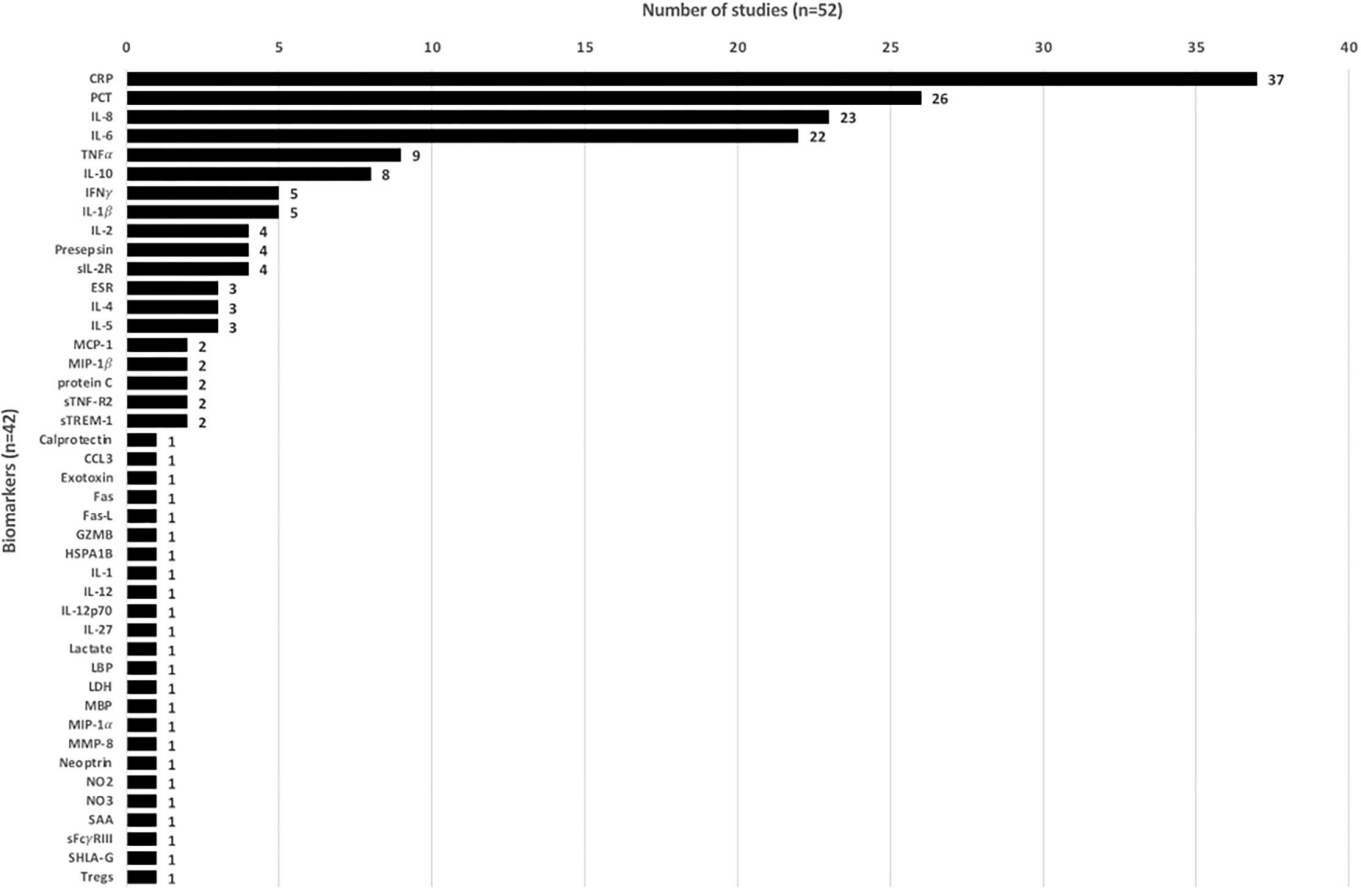
Assessed biomarkers across the included studies (*n* = 47 studies).

Overall, 52 studies analyzed 43 biomarkers, of which 24 only were in one study each. Most of the studied biomarkers were CRP (*n* = 37, 71.2%), PCT (*n* = 26, 50.0%), interleukin (IL)-6 (*n* = 23, 44.2%), and IL-8 (*n* = 22, 42.3%) (Figure 2). In total, 47 studies assessed ≥1 biomarker in their respective study population.

### Quality and Bias Assessment

**Figure 3:**
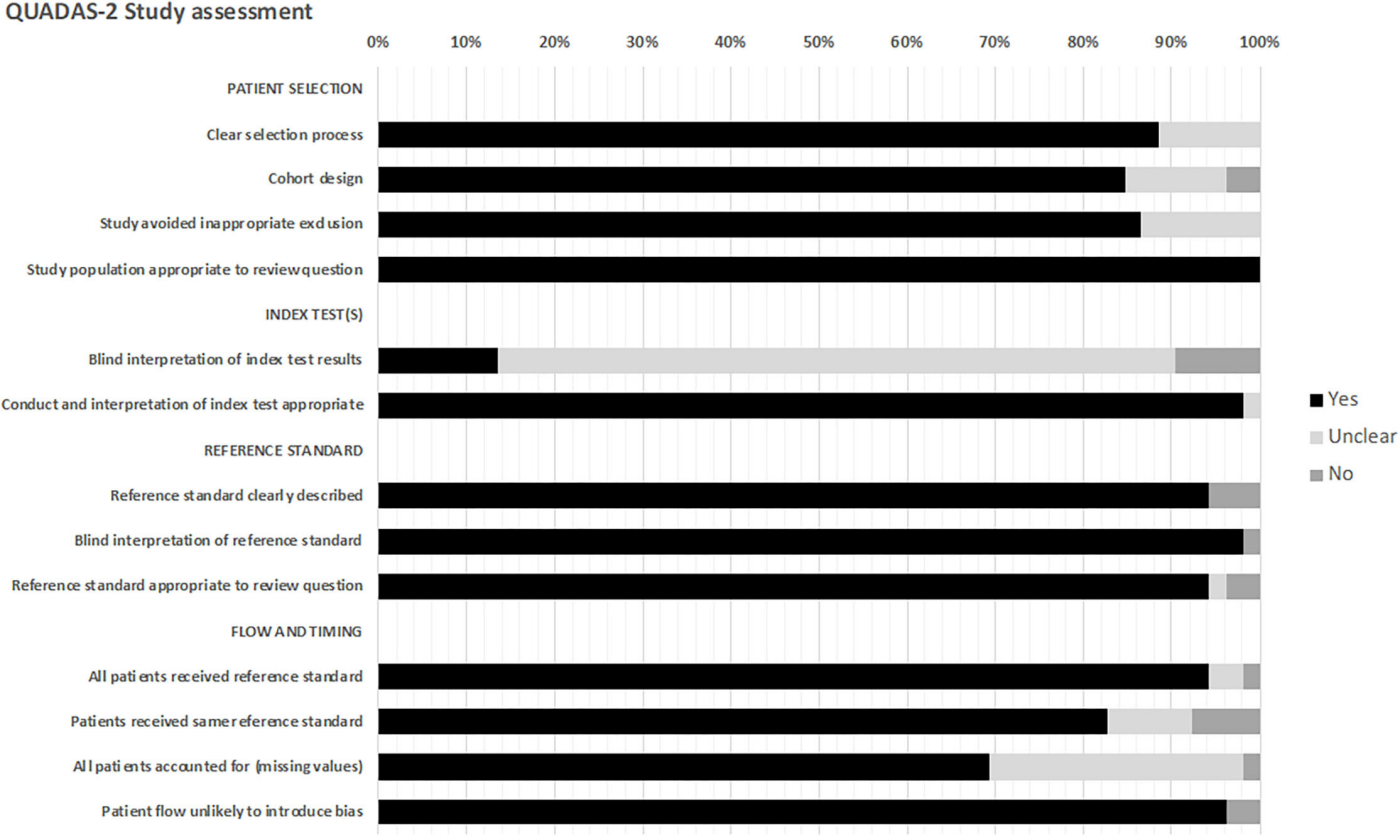
Overall summary of quality and bias assessment (*n* = 52 studies).

All studies underwent risk of bias assessment using QUADAS-2 (Figure 3, detailed in Supplementary Data 2).

Twenty-four studies were of fair quality (46.1%), 22 studies were good (42.3%), and six studies were poor (11.5%). The selection process was inadequately described in six studies, and six studies did not state if they were retrospective or prospective. Seven studies did not clearly state how inappropriate exclusions were avoided.

The majority of studies did not state if the index test results had blinded interpretation independent of the reference standard test (86.3%). The reference standards were clearly described.

Regarding “flow and timing,” in two studies (9, 33), it was unclear if all patients received the same reference standard. Fifteen studies did not state if all patients were accounted for. Two studies (9, 42) may have introduced patient flow bias, as one did not state the index test timepoints, and in one >50% of patients did not receive the index test at the prespecified timepoint.

### Biomarker Assessment for Predicting Bacterial Infection at Admission

Eighteen biomarkers were not discriminatory at admission for the prediction of bacterial infection. For Tregs (27), Neoptrin (40), and IL-12 (26), only one study each found a significant association between the increased levels of the respective biomarker and bacterial infection. Four biomarkers were assessed in a combined model in one study (45) and were excluded from further analysis as the available literature is too limited for effective comparison.

For 22 biomarkers, significant associations were described between biomarker and predicting bacterial infection at admission (Table 2). CRP, PCT, IL-6, IL-8, tumor necrosis factor alpha (TNFα), and IL-10 were the most studied biomarkers and had ≥2 studies statistically analyzing their predictive performance.

**Table 2:** Biomarker performance for biomarkers significantly associated with bacterial infection at admission.

**Biomarker**	**Studies (*n*)**	**Study size**	**Significant**	**Cutoff**	**% sensitivity**	**% specificity**	**Countries (*n*)**
		**[episodes, range (median)]**	**results (*n*)**	**range**	**(range)**	**(range)**	
CRP	37	31–3,118 (87.5)	16/37	2.5–105 mg/L	24.2–100	15.5–87.3	17
PCT	26	33–3,118 (90)	23/26	0.25–2.86 ng/ml	12–94	42.8–94.3	15
IL-8	23	31–3,023 (61)	20/23	30–320 pg/ml	49–92	54–100	11
IL-6	22	41–3,118 (72.5)	17/22	41–1,000 pg/ml	51.0–100	34.7–96	13
TNFα	9	27–3,118 (72)	3/9	≥5.0 pg/ml	45.1	92.3	7
IL-10	9	41–3,118 (58.5)	7/9	≥5.04–50 pg/ml	51.2–92.9	44.4–92	7
IFNγ	5	27–3,118 (100)	3/5	–	–	–	3
IL-1β	5	48–221 (61)	0/5	–	–	–	5
IL-2	4	27–3,023 (79)	1/4	–	–	–	3
Presepsin	4	39–90 (54.5)	2/4	>951 pg/ml	93.8	100	4
sIL-2R	4	48–62 (55.5)	1/4	1,658 ng/L	77	67	3
ESR	3	58–735 (60)	1/3	–	–	–	2
IL-4	3	58–3,023 (100)	0/3	–	–	–	2
IL-5	3	31–58 (55)	1/3	8 pg/dl	67	96	2
MCP-1	2	58–85	2/2	1,650–35,000 pg/ml	70–80	62–82	2
Fas	1	72	1	<100 pg/ml	90	37	1
Fas-L	1	72	1	200 pg/ml	90	30	1
IL-12	1	31	1	–	–	–	1
IL-27	1	400	1	≥2.0 ng/ml	37	79	1
Lactate	1	100	1	≥1.8 mmol/l	87	75	1
LBP	1	90	1	>41.8 ng/L	61.1	76.8	1
LDH	1	152	1	≥298.5 U/L	70.6	60.2	1

Erythrocyte sedimentation rate, interferon gamma, IL-1β, IL-2, and, IL-4 were studied in multiple studies and found to have elevated levels associated with bacterial infection compared to controls or non-bacterial infection, but no performance analysis was described (27, 30–32, 38, 41, 60, 66, 67, 72–74). For presepsin, soluble IL-2 receptor, IL-5, and monocyte chemotactic protein-1, multiple studies did assess the potential for predicting bacterial infection, but only one study per biomarker stated the sensitivity and specificity of data (Table 2).

#### CRP

CRP is an acute phase inflammatory cytokine induced by IL-6. The concentrations during an infection depend on the extent of tissue destruction, presence of malignant disease, and fever duration. It increases in the first 24–48 h after the onset of inflammation. It is known for its poor specificity (10, 12).

Thirty-seven studies assessed CRP as a biomarker for bacterial infection for HR children. Sixteen of these (41.6%) found significant associations between elevated CRP and bacterial infection, either microbiologically or clinically confirmed. Moreover, 14/16 significant studies provided predictive data with various cutoff values (range: 5–120 mg/L) (6, 9, 31, 33, 35, 37, 39, 40, 48, 53, 55, 62, 63, 74).

The low cutoff values provided good sensitivity (77.7–92.3%) but poor specificity (15.5–72.2%). The higher cutoff values saw a decrease of sensitivity (24.2–77.8%) and a slight increase of specificity (63–87.3%), yet the values were subpar to be an effective marker.

One study (9) reported 100% sensitivity for a CRP cutoff of 41.2 mg/L, but with 64.3% specificity. As a biomarker for bacterial infection in high-risk children, CRP remains a poor predictor.

#### PCT

PCT is a 116-amino-acid peptide with the same sequence as the prohormone of calcitonin. Considered an acute-phase protein, the PCT levels are low in healthy individuals. PCT is a stable molecule, and the levels rapidly increase in severe (bacterial) infections, without changing the calcitonin levels. In infections in the immunocompetent, PCT is known to increase earlier than CRP, usually within 2–4 h after onset and with peak levels after 24–48 h in septic patients.

Twenty-six studies assessed PCT for the prediction of bacterial infection, of which 23 found significant associations between increased PCT levels at admission in bacterial infection. Furthermore, 17/23 studies (73.9%) provided predictive data with cutoff values ranging 0.25–2.86 ng/ml. Across the studies, seven different measurement techniques were used. Eighteen used immunoassays, of which most were enzyme-linked immunosorbent assays (ELISA) and LUMItest, at seven and six times, respectively. Two studies used flow cytometry, and one used time-resolved amplified cryptate emission. The cutoff values among PCT studies were variable. The sensitivity ranged between 12 and 94%, with the highest sensitivities reported with a cutoff of 0.25 (72) or 2.0 ng/ml (41). Although the range is smaller (42.8–96.5%), the specificity of PCT turned out to have mixed performance across the cutoff values. A specificity of 94.2% was reported for a cutoff value of 0.3 ng/ml (66), and a specificity of 96.5% was reported for a cutoff of 2.0 ng/ml (41). Lowest specificities of 42.8 and 50% were reported on cutoff values of 0.25 and 0.38 ng/ml (71, 72).

#### IL-8

IL-8 is a pro-inflammatory cytokine released from monocytes, endothelial cells, and fibroblasts. Production is induced by IL-1, TNFα, and lipopolysaccharide-binding protein. The IL-8 levels increase more quickly than CRP and are reported to rise before fever onset (75, 76).

Twenty-two studies assessed IL-8, of which 19 found a significant association between increased IL-8 levels and infection. Five techniques for IL-8 measurement were used across the studies. The most common were ELISA in 12/22 studies, followed by five studies utilizing flow cytometry. In total, 9/22 studies analyzed IL-8's predictive properties, with cutoff values ranging 30–320 pg/ml. The sensitivities reported were similar across the cutoff values (56–92%), and the specificity tended to be higher if a higher cutoff value was used, with a cutoff of 30 pg/ml showing specificity of 59% (36) and a cutoff value of 320 pg/ml yielding 79–100% specificity.

#### IL-6

IL-6 is a pleiotropic cytokine produced by T-lymphocytes, macrophages, and endothelial cells. Synthesis and secretion are stimulated by IL-1. It influences multiple processes: upregulating neutrophil production, B-lymphocyte proliferation, and acute phase protein synthesis induction. It plays a vital role in trans-signaling in sepsis pathogenesis, and the levels peak early in the illness (26).

Twenty-two studies analyzed IL-6 use. In total, 17/22 studies (77.3%) reported significant associations between elevated IL-6 and bacterial infection. Six measurement techniques were utilized, the most common being ELISA in 11/22 studies. Of 17 studies with significant associations, 12 reported predictive data with cutoff values ranging 41.8–1,000 pg/ml. Higher cutoffs provided higher specificity, and lower cutoffs provided higher sensitivity. IL-6 sensitivity ranged 50–100%, and the specificity was 34.7–96%. Moreover, 100% sensitivity was reported with 60-pg/ml cutoff; however, the specificity was 34.7% (72). A cutoff of 235 pg/ml provided the best specificity at 91%, with a sensitivity of 89% (50).

#### TNFα

TNFα, a small cytokine secreted by macrophages and other antigen-presenting cells, induces immune response by stimulating T-lymphocyte activation. It subsequently promotes humoral and cellular immune responses *via* cell signaling (77).

Nine studies assessed TNFα, and three found significant associations between the elevated levels of TNFα and bacterial infection. Only one study calculated the predictive data, reporting a sensitivity of 45.1% and specificity of 92.3% using a cutoff value of 5.0 pg/ml (73).

#### IL-10

IL-10, an anti-inflammatory cytokine produced by macrophages and T-helper lymphocytes, plays an essential role in limiting the inflammatory process and preventing excessive host damaging (78).

Nine studies analyzed IL-10 as a predictive biomarker, with six studies reporting significant associations between the elevated IL-10 levels and bacterial or severe infection. Immunoassays were used in four and flow cytometry in five. In total, five different reported diagnostic measurement techniques were used.

Two studies report the predictive data. Sahbudak et al. (62) reported a sensitivity of 92.9% and specificity of 44.4% for a cutoff of 5.04 pg/ml in predicting a bacterial infection.

Xia et al. (31) calculated the predictive performance for IL-10 and gram-negative bacteremia and for IL-10 and severe infection. At a cutoff of 32.3 pg/ml, IL-10 had optimal sensitivity for predicting gram-negative bacteremia at 69.2% and optimal specificity for a cutoff of 100 pg/ml at 84.8%.

For severe infection, highest sensitivity and specificity were calculated at a cutoff of 49.5 pg/ml at 77.6 and 94.9% respectively.

### Biomarker Assessment for Viral and Fungal Infection at Admission

Twelve studies reported on biomarkers for viral infection prediction, and 10 studies reported on fungal infection (Table 1). For viral infection, two studies reported significantly elevated levels for IL-6 (38), and one study reported significantly elevated CRP (31, 34), but the numbers were too low to draw conclusions.

For fungal infections, one study (34) found increased PCT and CRP levels in children with fungal infection. There was no further statistical analysis performed regarding performance.

### Comparison of Biomarkers for Predicting Bacterial Infection

Mostly compared were CRP and PCT, with eight studies comparing their performance. Five of these found PCT to be performing better (34, 40, 41, 65, 72), one found CRP (63) to be performing better, and two studies found neither to be outperforming (6, 46).

CRP was also compared to IL-6 (6, 36, 46, 68, 72, 74), IL-8 (36, 46, 63, 64), and IL-10 (74). CRP did not outperform these ILs in any of these studies. IL-6, IL-8, and IL-10 outperformed CRP in 3/6 studies. In the remaining studies, both biomarkers performed equally.

The comparative performance of PCT and ILs was explored in six studies for IL-6 (6, 13, 31, 41, 72, 74), 5 studies for IL-8 (13, 41, 46, 63, 72), and two studies for IL-10 (31, 74). Two studies found IL-6 and two found PCT to be better compared to each other, respectively, and in two they performed equally. For IL-8 and PCT, it showed a similar mixed picture. The two studies comparing PCT and IL-10 were in favor of IL-10. Comparisons IL-6 and IL-8 were reported in five studies (36, 46, 62, 67, 72), with two favoring IL-6 and one favoring IL-8, and two could not state a better biomarker.

## Discussion

This review analyzed 51 studies, including 43 biomarkers for infection prediction in immunocompromised children. We illustrate the ongoing quest for better diagnostics for fever in this vulnerable group and did not limit to patients presenting with febrile neutropenia, aiming to reflect clinical practice more truthfully.

In total, 39.5% of the studied biomarkers were not discriminatory between bacterial and non-bacterial causes of febrile illness. Literature assessing biomarker performance in predicting viral or fungal disease was sparse, although potentially equally important causes of a severe illness in this group (79).

Most studies were performed in children with malignancy. HSCT, PID, and solid organ transplant patients are underrepresented.

Across studies, the definitions of fever and neutropenia were quite consistent, with recent studies providing more consistent definitions, thus allowing a better comparison.

The differences between the groups and definitions used lead to heterogeneity—for example, bacteremia, sepsis, severe infection, microbiologically or clinically documented infection, localized infection, sepsis, and bacteremia were used, which are not equally interchangeable. In biomarker predictive performance assessment, we observed that, not unexpectedly, by increasing the cutoff levels, the sensitivities decreased and the specificities increased. The literature shows that many biomarkers have no normal range of cutoff value identified. The techniques used for biomarker measurement varied across the studies, complicating the interpretation of cutoff values for biomarker performance, especially for PCT, as 7 different methods of measurement were used.

Of all biomarkers assessed, CRP, PCT, and IL-6/8/10 were the most thoroughly studied. In studies comparing PCT and CRP, PCT was felt superior to CRP for predicting bacterial infection (2, 42, 50, 64). Kitanovski et al. (6) found that PCT in this group performed well in predicting systemic bacterial infections, but not for focal infections.

In HSCT patients, an elevated PCT level is also associated with other transplant-related adverse events (34, 38).

The literature shows a tendency to favor IL-6 and IL-8 over CRP, having better specificities.

There is insufficient evidence for these markers to replace CRP in current practice. Most studies also assessed the use of biomarkers for discriminating between non-bacterial and bacterial infection. The role of inflammatory illness has not yet been well-explored in the HR population. IL-6 might have a role in the prediction of gram-negative bacterial infection in HR patients, as both studies in cancer patients and HSCT patients describe significantly higher IL-6 levels compared to CRP and IL-8 (31, 43, 50, 67, 73, 74). Similar statements are made for IL-8 and performance in the prediction of gram-negative infection (49, 62, 67).

Although many studies have been conducted on this topic, generalizability remains a challenge due to methodological inconsistency. Definitions changed over time for important features of febrile illness in this group, e.g., fever and neutropenia, although more methodological consistency is observed in recent literature. Furthermore, the outcome measures would benefit from more uniformity. We observed a variety of outcomes in the studies, which are similar but not interchangeable, such as bacteremia and sepsis, or which are less commonly used, such as clinically documented infection. We acknowledge that this will be challenging, as clinical definitions in the field of febrile illness are still a topic of debate.

Novel diagnostics, e.g., host RNA biosignatures, have proven able to accurately differentiate between viral and bacterial infections in immunocompetent children, with sensitivities of 94–98% and specificities of 91–96% (11, 12). It is unknown if a similar approach is feasible for immunocompromised HR children, particularly those with low cell counts.

In conclusion, this review shows that adequately diagnosing febrile illness in immunocompromised children at ED remains challenging. Better diagnostics are needed for this population.

## Data Availability Statement

The raw data supporting the conclusions of this article will be made available by the authors, without undue reservation.

## Author Contributions

FV, AG, and ME wrote the manuscript. FV was responsible for the literature search, search strategies, study selection, data extraction, bias assessment, and analysis. ME and AG reviewed and supervised FV during these processes. All co-authors reviewed the manuscript and agreed to its submission for publication.

## Funding

FV and ME were funded by the European Union's Horizon 2020 research and innovation program, under grant agreement numbers 668303 and 848196.

## Conflict of Interest

The authors declare that the research was conducted in the absence of any commercial or financial relationships that could be construed as a potential conflict of interest.

## Publisher's Note

All claims expressed in this article are solely those of the authors and do not necessarily represent those of their affiliated organizations, or those of the publisher, the editors and the reviewers. Any product that may be evaluated in this article, or claim that may be made by its manufacturer, is not guaranteed or endorsed by the publisher.

## Supplementary Material

Supplementary Data 1 Search strategy for Medline.

Supplementary Data 2 In-depth QUADAS-2 assessment for each study.
