# Supplementary material for: Biomarkers for Diagnosing Febrile Illness in Immunocompromised Children: A Systematic Review of the Literature
Source: Front Pediatr. 2022 Mar 10;10:828569. doi: 10.3389/fped.2022.828569 (PMC8965604; doi:10.3389/fped.2022.828569)
Supplement: Supplementary Data 1 — Search strategy for Medline. [file Data_Sheet_1.DOCX]

**Supplementary data 1**

**MEDLINE and MEDLINE and In-Process & Other Non-Index Citations Search Strategy**

1 Infant/ (801032)

2 Child, Preschool/ or Child/ (1936156)

3 Adolescent/ (2058059)

4 Neoplasms/ (431101)

5 Primary Immunodeficiency Diseases/ or Severe Combined Immunodeficiency/ (4454)

6 Immunologic Deficiency Syndromes/ (14924)

7 Febrile Neutropenia/ or Chemotherapy-Induced Febrile Neutropenia/ or Neutropenia/ (18879)

8 Transplants/ (5271)

9 Bone Marrow Transplantation/ (44769)

10 Hematopoietic Stem Cell Transplantation/ (43025)

11 Fever/ (39960)

12 Biomarkers/ (287188)

13 Cytokines/ (151664)

14 Infections/ (39433)

15 Bacterial Infections/ (71068)

16 Virus Diseases/ (38870)

17 Mycoses/ (22915)

18 Bacteremia/ (25276)

19 Sepsis/ (60648)

20 1 or 2 or 3 (3295297)

21 4 or 5 or 6 or 7 or 8 or 9 or 10 (548412)

22 12 or 13 (429336)

23 11 or 14 or 15 or 16 or 17 or 18 or 19 (275155)

24 20 and 21 (70095)

25 22 and 23 (10439)

26 24 and 25 (143)
