# Supplementary material for: Biomarkers for Diagnosing Febrile Illness in Immunocompromised Children: A Systematic Review of the Literature
Source: Front Pediatr. 2022 Mar 10;10:828569. doi: 10.3389/fped.2022.828569 (PMC8965604; doi:10.3389/fped.2022.828569)
Supplement: Supplementary Data 2 — In-depth QUADAS-2 assessment for each study. [file Data_Sheet_2.DOCX]

**Overview of QUADAS-2 risk of bias assessment**

Using QUADAS-2 articles are judged for bias and applicability on four domains: patient selection, index test(s), reference standard, and, flow and timing. Patient selection focuses potential bias introduction on study design, recruitment and study population. Index tests focuses on potential bias by how the index test is interpret and conducted. Reference standard focuses on bias introduced by usage of reference standard test, if they can adequately diagnoses the condition and if it is interpret independently from the index test. Flow and timing focuses on potential introduction of bias by patient flow (did all patients receive all tests? Was there an interval between the tests? Is there missing data?)

After assessment study quality is deemed good (no ‘high’ risk of bias, max. 2 unclears), fair (max. 1 ‘high’ risk of bias) or poor (>1 ‘high’ risk of bias)

| Study | Patient Selection | Index Test(s) | Reference Standard | Flow and Timing | Overall Study Quality (good, fair, poor) |
| --- | --- | --- | --- | --- | --- |
| Aquino, 2012 | Risk of bias: unclear   - Selection process unclear - Prospective cohort - Clear definitions used - Avoided inappropriate exclusion   Concerns about applicability: low  Appropriate study population | Risk of bias: unclear   - Unclear if index tests interpreted with knowledge of outcome - Unclear if thresholds prespecified   Concerns about applicability: low   - Execution and conduct of tests clear | Risk of bias: low   - Same outcome assessed for all biomarkers - Outcome assessment independent of test - Adequate reference standard description   Concerns about applicability: low  Relevant outcomes | Risk of bias: unclear   - All patients included had outcome assessed - Test conduct clear on predetermined timepoint (prior to antibiotics) - Unclear if missing data   Concerns about flow introduced bias: unclear | Fair |
| Ashkenazi-Hoffnung, 2017 | Risk of bias: low  - Selection process clear  - Retrospective cohort  - Avoided inappropriate exclusion  - Clear definitions used  - Infections clearly defined  Concerns about applicability: low  - Appropriate study population | Risk of bias: high  - Not reported if index tests were interpreted with knowledge of outcome  - threshold was not pre-specified  Concerns about applicability: low  - Execution, conduct and interpretation of tests clear | Risk of bias: high  - Same outcome assessed for all biomarkers  - Outcome assessment independent of test  - Inadequate reference standards description  Concerns about applicability: low  - Relevant outcomes | Risk of bias: low  - All patients outcome assessed  - Missing data clearly stated  - Tests conducted at ED admission, unclear if prior to antibiotics  Concerns patient flow introduced bias: low | Fair |
| Badurdeen, 2012 | Risk of bias: unclear  - Selection process clear  - Prospective cohort  - Clear definitions used  - Age of paediatric patients unclear  - Exclusion criteria not stated  - Unclear if inappropriate exclusion  Concerns about applicability: low  - Appropriate study population | Risk of bias: low   - Index tests interpreted without knowledge of outcome - Thresholds prespecified   Concerns about applicability: low  Execution, conduct and interpretation of tests clear | Risk of bias: low   - Same outcome assessed for all biomarkers - Outcome assessment independent of test - Adequate reference standard description   Concerns about applicability: low  Relevant outcomes | Risk of bias: unclear   - All patients included had outcome assessed - Missing data on some cytokines, unclear why - Some data not reported as ‘non-contributory’   Concerns about flow introduced bias: low | Good |
| Baraka, 2018 | Risk of Bias: high  - Selection process clear  - Case-control study  - Avoidance of inappropriate exclusion: unclear  Concerns about applicability: low  - Appropriate study population | Risk of bias: unclear - Unclear if index test interpreted without knowledge of outcome  - Threshold not pre-specified  Concerns about applicability: Low  - Execution, conduct and interpretation of testes clear. | Risk of bias: high  - Same outcome assessed for all biomarkers  - Outcome assessment independent of test  - Inadequate reference standard description  Concerns about applicability: low  - Relevant outcomes | Risk of bias: high  - All patients outcome assessed  - No missing data  - Tests conducted in all patients but at which point in the clinical course is not defined, nor if prior to antibiotics.  Concerns patient flow introduced bias: high | Poor |
| Cabanillas Stanchi, 2019 | Risk of bias: low  - Selection process clear  - Retrospective study with consecutively enrolled cohort  - Clear definitions used  - Avoided inappropriate exclusion  Concerns about applicability: low  - Appropriate study population | Risk of bias: high  - Index tests were interpreted with knowledge of outcome  - Threshold prespecified.  Concerns about applicability: low  - Execution, conduct and interpretation of testes clear. | Risk of bias: low  - Same outcome assessed for all biomarkers  - Outcome assessment independent of test  - Adequate reference standards description.  Concerns about applicability: low  - Relevant outcomes | Risk of bias: low  - All patients outcome assessed  - No missing data  - Test conduct clearly defined. All patients on prophylactic antibiotics.  Concerns patient flow introduced bias: low | Good |
| Delebarre, 2015 | Risk of bias: low  - Selection process clear  - Retrospective cohort  - Clear definitions used  - Avoided inappropriate exclusion  Concerns about applicability: low  - Appropriate study population | Risk of bias: unclear   - Unclear if index tests interpreted with knowledge of outcome - Thresholds prespecified   Concerns about applicability: unclear  Execution, conduct and interpretation of tests unclear | Risk of bias: low   - Same outcome assessed for all biomarkers - Outcome assessment independent of test - Adequate reference standard description   Concerns about applicability: unclear  Not clear if reported death/severe sepsis cases were included in the respective group analysis | Risk of bias: low   - All patients included had outcome assessed - Missing data clearly stated. - Test conduct clear on predetermined timepoint   Concerns about flow introduced bias: low | Good |
| Diepold, 2008 | Risk of bias: low  - Selection process clear  - Prospective cohort  - Clear definitions used  - Avoided inappropriate exclusion  Concerns about applicability: low  - Appropriate study population | Risk of bias: unclear   - Unclear if index tests interpreted without knowledge of outcome - Thresholds prespecified   Concerns about applicability: low  Execution, conduct and interpretation of tests clear | Risk of bias: low   - Same outcome assessed for all biomarkers - Outcome assessment independent of test - Adequate reference standard description   Concerns about applicability: low  Relevant outcomes | Risk of bias: low   - Most patients outcome assessed, clearly stated excluded episodes and why - Missing data clearly stated - Test conduct clear on predetermined timepoint   Concerns about flow introduced bias: low | Good |
| Doerflinger, 2021 | Risk of bias: low   - Selection process clear - Prospective cohort part of larger study - Clear definitions used - Avoided inappropriate exclusion   Concerns about applicability: low   - Appropriate study population | Risk of bias: unclear   - Unclear if index tests interpreted with knowledge of outcome - Thresholds prespecified   Concerns about applicability: low  Execution, conduct and interpretation of tests clear | Risk of bias: low   - Same outcome assessed for all biomarkers, only relevant cytokines used for in-depth analysis - Outcome assessment independent of test - Adequate reference description   Concerns about applicability: low  Relevant outcomes | Risk of bias: low   - All patients outcome assessed, stated clearly. - No missing data, as patients already excluded as per exclusion criteria - Test conduct clear and on predetermined timepoint   Concerns about flow introduced bias: low | Good |
| Döring, 2015 | Risk of bias: low   - Selection process clear - Prospective cohort - Clear definitions used - Avoided inappropriate exclusion   Concerns about applicability: low  Appropriate study population | Risk of bias: high   - Index tests interpreted with knowledge of outcome - Thresholds not prespecified   Concerns about applicability: low  Execution, conduct and interpretation of tests clear | Risk of bias: low   - Same outcome assessed for all biomarkers - Outcome assessment independent of test - Adequate reference standard description   Concerns about applicability: low  Relevant outcomes | Risk of bias: low   - All patients outcome assessed, clear exclusion criteria - No missing data - Test conduct clear on predetermined timepoint   Concerns about flow introduced bias: low | Fair |
| El-Maghraby, 2007 | Risk of bias: low  - Selection process clear  - Prospective cohort  - Clear definitions used  - Avoided inappropriate exclusion  Concerns about applicability: low  - Appropriate study population | Risk of bias: unclear   - Unclear if index tests interpreted with knowledge of outcome - Thresholds prespecified   Concerns about applicability: low  Execution, conduct and interpretation of tests clear | Risk of bias: low   - Same outcome assessed for all biomarkers - Outcome assessment independent of test - Adequate reference standard description   Concerns about applicability: low   - Relevant outcomes | Risk of bias: low   - All patients included had outcome assessed - No missing data - Test conduct clear on predetermined timepoint   Concerns about flow introduced bias: low | Good |
| Gunasekaran, 2016 | Risk of bias: unclear  - Selection process unclear  - Prospective cohort  - Clear definitions used, except for age definition for a child  - Unclear if avoided inappropriate exclusion  Concerns about applicability: low  - Appropriate study population | Risk of bias: unclear   - Unclear if index tests interpreted with knowledge of outcome - Thresholds prespecified   Concerns about applicability: low  Execution, conduct and interpretation of tests clear | Risk of bias: low   - Same outcome assessed for all biomarkers - Outcome assessment independent of test - Adequate reference standard description   Concerns about applicability: low  Relevant outcomes | Risk of bias: low   - All patients included had outcome assessed - Clearly described excluded episodes due to incomplete data - Test conduct clear on predetermined timepoint   Concerns about flow introduced bias: low | Good |
| Hatzistilianou, 2007 | Risk of bias: low  - Selection process clear  - Prospective cohort  - Clear definitions used  - Avoided inappropriate exclusion  Concerns about applicability: low  - Appropriate study population | Risk of bias: unclear   - Unclear if index tests interpreted with knowledge of outcome - Thresholds prespecified   Concerns about applicability: low  Execution, conduct and interpretation of tests clear | Risk of bias: low   - Same outcome assessed for all biomarkers - Outcome assessment independent of test - Adequate reference standard description   Concerns about applicability: low  Relevant outcomes | Risk of bias: unclear   - All patients outcome assessed, - Unclear if missing data - Test conduct clear on predetermined timepoint   Concerns about flow introduced bias: low | Fair |
| Hatzistilianou, 2010 | Risk of bias: unclear  - Selection process clear  - Unclear study design, not stated if retro- or prospective  - Clear definitions used  - Avoided inappropriate exclusion  Concerns about applicability: low  - Appropriate study population | Risk of bias: unclear   - Unclear if index tests interpreted without knowledge of outcome - Thresholds prespecified   Concerns about applicability: low  Execution, conduct and interpretation of tests clear | Risk of bias: low   - Same outcome assessed for all biomarkers - Outcome assessment independent of test - Adequate reference standard description   Concerns about applicability: low  Relevant outcomes | Risk of bias: low   - All patients outcome assessed, clear exclusion criteria - No missing data - Test conduct clear on predetermined timepoint   Concerns about flow introduced bias: low | Fair |
| Hazan, 2014 | Risk of bias: unclear  - Selection process clear  - Prospective cohort  - Clear definitions used  - Unclear exclusion criteria  - Unclear if avoided inappropriate exlusion  Concerns about applicability: low  - Appropriate study population | Risk of bias: unclear   - Unclear if index tests interpreted with knowledge of outcome - Thresholds prespecified   Concerns about applicability: unclear  Execution, conduct and interpretation of tests unclear | Risk of bias: low   - Same outcome assessed for all biomarkers - Outcome assessment independent of test - Adequate reference standard description   Concerns about applicability: low   - Relevant outcomes | Risk of bias: high   - All patients included had outcome assessed - >50% missing index test value - Unclear timing of serial testing.   Concerns about flow introduced bias: high | Poor |
| Heney 1992 | Risk of bias: unclear  - Selection process clear  - Prospective study  - Unclear definition of neutropenia.  - Avoided inappropriate exclusion  Concerns about applicability: low  - Appropriate study population | Risk of bias: unclear   - Unclear if index tests interpreted without knowledge of outcome   Concerns about applicability: low  Execution, conduct and interpretation of tests clear | Risk of bias: low   - Same outcome assessed for all biomarkers - Outcome assessment independent of test - Adequate reference standard description   Concerns about applicability: low  Relevant outcomes | Risk of bias: unclear   - All patients outcome assessed, clear exclusion criteria - Unclear if missing data - Test conduct clear on predetermined timepoints   Concerns about flow introduced bias: low | Fair |
| Hodge, 2006 | Risk of bias: unclear  - Selection process unclear  - Unclear if restrospective/prospective  - Clear definitions used  - Avoided inappropriate exclusion  Concerns about applicability: low  - Appropriate study population | Risk of bias: unclear   - Unclear if index tests interpreted with knowledge of outcome - Thresholds prespecified   Concerns about applicability: low  Execution, conduct and interpretation of tests clear | Risk of bias: low   - Same outcome assessed for all biomarkers - Outcome assessment independent of test - Adequate reference standard description   Concerns about applicability: low  Relevant outcomes | Risk of bias: unclear   - Unclear if all patients outcome assessed, - No missing data - Test conduct clear on predetermined timepoint   Concerns about flow introduced bias: low | Poor |
| Hodge, 2011 | Risk of bias: low  - Selection process clear  - Prospective cohort  - Clear definitions used  - Avoided inappropriate exclusion  Concerns about applicability: low  - Appropriate study population | Risk of bias: high   - Index tests interpreted with knowledge of outcome - Unclear if thresholds prespecified   Concerns about applicability: low  Execution, conduct and interpretation of tests clear | Risk of bias: low   - Same outcome assessed for all biomarkers - Outcome assessment independent of test - Adequate reference standard description   Concerns about applicability: low  Relevant outcomes | Risk of bias: low   - All patients outcome assessed, - No missing data - Test conduct clear on predetermined timepoint   Concerns about flow introduced bias: low | Fair |
| Jacobs, 2018 | Risk of bias: low  - Selection process clear  - Prospective cohort  - Clear definitions used  - Avoided inappropriate exclusion  Concerns about applicability: low  - Appropriate study population | Risk of bias: low  - index tests interpreted without knowledge of outcome.  - threshold pre-specified  Concerns about applicability: low  - Execution, conduct and interpretation of tests clear | Risk of bias: low  - Same outcome assessed for all biomarkers  - Outcome assessment independent of test  - Adequate reference standards description  Concerns about applicability: low  - Relevant outcomes | Risk of bias: low  - All patients outcome assessed  - No missing data  - Tests conducted <24h of presentation, corrected for antibiotics in previous 24h  Concerns patient flow introduced bias: low | Good |
| Jacobs, 2019 | Risk of bias: low   - Selection process clear - Prospective cohort - Clear definitions used - Avoided inappropriate exclusion   Concerns about applicability: low  Appropriate study population | Risk of bias: low   - Index tests interpreted without knowledge of outcome - Thresholds prespecified   Concerns about applicability: low  Execution, conduct and interpretation of tests clear | Risk of bias: low   - Same outcome assessed for all biomarkers - Outcome assessment independent of test - Adequate reference standard description   Concerns about applicability: low  Relevant outcomes | Risk of bias: low   - All patients outcome assessed, clear exclusion criteria - No missing data - Test conduct clear on predetermined timepoint   Concerns about flow introduced bias: low | Good |
| Karakurt, 2014 | Risk of bias: low  - Selection process clear  - Prospective cohort  - Clear definitions used  - Avoided inappropriate exclusion  Concerns about applicability: low  - Appropriate study population | Risk of bias: high   - Unclear if index tests interpreted without knowledge of outcome - Thresholds not prespecified   Concerns about applicability: low  Execution, conduct and interpretation of tests clear | Risk of bias: high   - Same outcome assessed for all biomarkers - Outcome assessment independent of test - Inadequate reference standard description   Concerns about applicability: low  Relevant outcomes | Risk of bias: low   - All patients outcome assessed, clear exclusion criteria - No missing data - Test conduct clear on predetermined timepoint   Concerns about flow introduced bias: low | Poor |
| Kassam, 2009 | Risk of bias: low  - Selection process clear  - Prospective cohort  - Part of a larger study, with different hypotheses.  - Clear definitions used  - Avoided inappropriate exclusion  Concerns about applicability: low  - Appropriate study population | Risk of bias: unclear   - Unclear if index tests interpreted with knowledge of outcome - Thresholds prespecified   Concerns about applicability: low  Execution, conduct and interpretation of tests clear | Risk of bias: low   - Same outcome assessed for all biomarkers - Outcome assessment independent of test - Adequate reference standard description   Concerns about applicability: low   - Relevant outcomes | Risk of bias: unclear   - All patients outcome assessed, - Unclear if missing data - Test conduct clear on predetermined timepoint   Concerns about flow introduced bias: low | Fair |
| Kitanovski, 2006 | Risk of bias: unclear  - Selection process clear  - Prospective cohort  - Unclear fever definition  - Avoided inappropriate exclusion  Concerns about applicability: low  - Appropriate study population | Risk of bias: unclear   - Unclear if index tests interpreted with knowledge of outcome - Thresholds prespecified   Concerns about applicability: low  Execution, conduct and interpretation of tests clear | Risk of bias: low   - Same outcome assessed for all biomarkers - Outcome assessment independent of test - Adequate reference standard description   Concerns about applicability: low  Relevant outcomes | Risk of bias: low   - All patients outcome assessed, - No missing data - Test conduct clear on predetermined timepoint   Concerns about flow introduced bias: low | Fair |
| Kitanovski, 2014 | Risk of bias: low  - Selection process clear  - Prospective cohort  - Clear definitions used  - Avoided inappropriate exclusion  Concerns about applicability: low  - Appropriate study population | Risk of bias: unclear   - Unclear if index tests interpreted without knowledge of outcome - Thresholds prespecified   Concerns about applicability: low  Execution, conduct and interpretation of tests clear | Risk of bias: low   - Same outcome assessed for all biomarkers - Outcome assessment independent of test - Adequate reference standard description   Concerns about applicability: low  Relevant outcomes | Risk of bias: low   - All patients outcome assessed, clear exclusion criteria - No missing data - Test conduct clear on predetermined timepoint   Concerns about flow introduced bias: low | Good |
| Lehrnbecher, 1999 | Risk of bias: low  - Selection process clear  - Prospective cohort  - Clear definitions used  - Avoided inappropriate exclusion  Concerns about applicability: low  - Appropriate study population | Risk of bias: unclear   - Unclear if index tests interpreted without knowledge of outcome - Unclear if thresholds prespecified   Concerns about applicability: low  Execution, conduct and interpretation of tests clear | Risk of bias: low   - Same outcome assessed for all biomarkers - Outcome assessment independent of test - Adequate reference standard description   Concerns about applicability: low  Relevant outcomes | Risk of bias: unclear   - All patients outcome assessed, clear exclusion criteria - Unclear if missing data - Test conduct clear on predetermined timepoints - Clearly stated why in depth analysis of 3 biomarkers was discontinued   Concerns about flow introduced bias: low | Fair |
| Lehrnbecher, 2004 | Risk of bias: unclear   - Selection process unclear - Unclear if retro-/prospective - Clear definitions used. - Avoided inappropriate exclusion   Concerns about applicability: low  Appropriate study population | Risk of bias: unclear   - Unclear if index tests interpreted with knowledge of outcome - Thresholds prespecified   Concerns about applicability: low  Execution, interpretation and conduct of tests clear | Risk of bias: low   - Same outcome assessed for all biomarkers - Outcome assessment independent of test - Adequate reference standard description   Concerns about applicability: low  Relevant outcomes | Risk of bias: low   - All patients included had outcome assessed - Test conduct clear on predetermined timepoint (prior to antibiotics) - No missing data   Concerns about flow introduced bias: low | Fair |
| Li, 2019 | Risk of bias: high  - Selection process clear  - Case-control study  - Clear definitions used  - Avoided inappropriate exclusion  Concerns about applicability: low  - Appropriate study population | Risk of bias: unclear   - Unclear if index tests interpreted with knowledge of outcome - Thresholds prespecified   Concerns about applicability: low  Execution, conduct and interpretation of tests clear | Risk of bias: low   - Same outcome assessed for all biomarkers - Outcome assessment independent of test - Adequate reference standard description   Concerns about applicability: low  Relevant outcomes | Risk of bias: unclear   - All patients outcome assessed, - Unclear if missing data - Test conduct clear on predetermined timepoint   Concerns about flow introduced bias: low | Fair |
| Martinez-Albarran, 2009 | Risk of bias: low  - Selection process clear  - Prospective cohort  - Clear definitions used  - Avoided inappropriate exclusion  Concerns about applicability: low  - Appropriate study population | Risk of bias: unclear   - Unclear if index tests interpreted with knowledge of outcome - Thresholds prespecified   Concerns about applicability: low  Execution, conduct and interpretation of tests clear | Risk of bias: low   - Same outcome assessed for all biomarkers - Outcome assessment independent of test - Adequate reference standard description   Concerns about applicability: low  Relevant outcomes | Risk of bias: low   - All patients outcome assessed, - No missing data, clearly stated why 4 eligible patients were excluded prior to analysis - Test conduct clear on predetermined timepoint   Concerns about flow introduced bias: low | Good |
| Miedema, 2011 | Risk of bias: low  - Selection process clear  - Prospective cohort  - Clear definitions used  - Avoided inappropriate exclusion  Concerns about applicability: low  - Appropriate study population | Risk of bias: unclear   - Unclear if index tests interpreted without knowledge of outcome - Thresholds prespecified   Concerns about applicability: low  Execution, conduct and interpretation of tests clear | Risk of bias: low   - Same outcome assessed for all biomarkers - Outcome assessment independent of test - Adequate reference standard description   Concerns about applicability: low  Relevant outcomes | Risk of bias: low   - All patients outcome assessed, clear exclusion criteria - No missing data - Test conduct clear on predetermined timepoints - Clearly stated why in depth analysis of 3 biomarkers was discontinued   Concerns about flow introduced bias: low | Good |
| Miedema 2014 | Risk of bias: unclear  - Selection process clear  - Prospective cohort  - Unclear definitions used  - Unclear if avoided inappropriate exclusion  Concerns about applicability: low  - Appropriate study population | Risk of bias: unclear   - Unclear if index tests interpreted without knowledge of outcome - Unclear if thresholds prespecified   Concerns about applicability: low  Execution, conduct and interpretation of tests clear | Risk of bias: low   - Same outcome assessed for all biomarkers - Outcome assessment independent of test - Adequate reference standard description   Concerns about applicability: low  Relevant outcomes | Risk of bias: low   - All patients outcome assessed, - No missing data - Test conduct clear on predetermined timepoint   Concerns about flow introduced bias: low | Fair |
| Nath, 2017 | Risk of bias: unclear  - Selection process clear  - Prospective cohort  - Unclear definition of fever or neutropenia  - Unclear if avoided inappropriate exclusion  Concerns about applicability: low  - Appropriate study population | Risk of bias: unclear   - Unclear if index tests interpreted with knowledge of outcome - Thresholds prespecified   Concerns about applicability: low  Execution, conduct and interpretation of tests clear | Risk of bias: low   - Same outcome assessed for all biomarkers - Outcome assessment independent of test - Adequate reference standard description   Concerns about applicability: low  Relevant outcomes | Risk of bias: low   - All patients outcome assessed, - No missing data - Test conduct clear on predetermined timepoint   Concerns about flow introduced bias: low | Good |
| Özdemir, 2019 | Risk of bias: unclear  - Selection process clear  - Unclear if pro-/retrospective  - Clear definitions used - Avoided inappropriate exclusion  Concerns about applicability: low  - Appropriate study population | Risk of bias: unclear  - Unclear if index tests interpreted without knowledge of outcome - Threshold prespecified  Concerns about applicability: low  - Excecution, conduct and interpretation of tests clear | Risk of bias: low  - Same outcome assessed for all biomarkers  - outcome assessment independent of test  - adequate reference standard description  Concerns about applicability: low  - relevant outcomes | Risk of bias: low  - All patients outcome assessed, clear exclusion criteria - missing data clearly stated  - Test conduct clear on predetermined timepoint  Concerns about flow introduced bias: low | Fair |
| Pacheco-Rosas, 2014 | Risk of bias: low  - Selection process clear  - Prospective cohort  - Clear definitions used  - Avoided inappropriate exclusion  Concerns about applicability: low  - Appropriate study population | Risk of bias: low   - Index tests interpreted without knowledge of outcome - Thresholds prespecified   Concerns about applicability: low  Execution, conduct and interpretation of tests clear | Risk of bias: low   - Same outcome assessed for all biomarkers - Outcome assessment independent of test - Adequate reference standard description   Concerns about applicability: low   - Relevant outcomes | Risk of bias: low   - All patients included had outcome assessed - No missing data - Did exclude 26 episodes, as they wanted to analyse 100 episodes - Test conduct clear on predetermined timepoint   Concerns about flow introduced bias: low | Good |
| Reyna-Figueroa, 2017 | Risk of bias: low   - Selection process clear - Prospective cohort - Clear definitions used - Avoided inappropriate exclusion   Concerns about applicability: low  Appropriate study population | Risk of bias: low   - Index tests interpreted without knowledge of outcome - Thresholds prespecified   Concerns about applicability: low  Execution, conduct and interpretation of tests clear | Risk of bias: low   - Same outcome assessed for all biomarkers - Outcome assessment independent of test - Adequate reference standard description   Concerns about applicability: low  Relevant outcomes | Risk of bias: low   - All patients outcome assessed, clear exclusion criteria - No missing data - Test conduct clear on predetermined timepoint   Concerns about flow introduced bias: low | Good |
| Riikonen 1992 | Risk of bias: low  - Selection process clear  - Prospective cohort  - Clear definitions used  - Avoided inappropriate exclusion  Concerns about applicability: low  - Appropriate study population | Risk of bias: unclear   - Unclear if index tests interpreted without knowledge of outcome - Thresholds prespecified   Concerns about applicability: low  Execution, conduct and interpretation of tests clear | Risk of bias: low   - Same outcome assessed for all biomarkers - Outcome assessment independent of test - Adequate reference standard description   Concerns about applicability: low  Relevant outcomes | Risk of bias: low   - All patients outcome assessed, - No missing data - Test conduct clear on predetermined timepoint   Concerns about flow introduced bias: low | Good |
| Riikonen, 1993 | Risk of bias: low  - Selection process clear  - Prospective cohort  - Clear definitions used  - Avoided inappropriate exclusion  Concerns about applicability: low  - Appropriate study population | Risk of bias: unclear   - Unclear if index tests interpreted without knowledge of outcome - Thresholds prespecified   Concerns about applicability: low  Execution, conduct and interpretation of tests clear | Risk of bias: low   - Same outcome assessed for all biomarkers - Outcome assessment independent of test - Adequate reference standard description   Concerns about applicability: low  Relevant outcomes | Risk of bias: unclear   - All patients outcome assessed, - Unclear if missing data - Test conduct clear on predetermined timepoint   Concerns about flow introduced bias: low | Good |
| Ruggiero, 2019 | Risk of bias: unclear  - Selection process clear  - Unclear if prospective or retrospective cohort  - Unclear age definition  - Avoided inappropriate exclusion  Concerns about applicability: low  - Appropriate study population | Risk of bias: unclear   - Unclear if index tests interpreted without knowledge of outcome - Thresholds prespecified   Concerns about applicability: low  Execution, conduct and interpretation of tests clear | Risk of bias: low   - Same outcome assessed for all biomarkers - Outcome assessment independent of test - Adequate reference standard description   Concerns about applicability: low  Relevant outcomes | Risk of bias: low   - All patients included had outcome assessed - Test conduct clear on predetermined timepoint (prior to antibiotics) - No missing data   Concerns about flow introduced bias: low | Fair |
| Şahbudak, 2017 | Risk of bias: low  - Selection process clear  - Prospective cohort  - Clear definitions used  - Avoided inappropriate exclusion  Concerns about applicability: low  - Appropriate study population | Risk of bias: Unclear   - Unclear if index tests interpreted without knowledge of outcome - Thresholds prespecified   Concerns about applicability: low  Execution, conduct and interpretation of tests clear | Risk of bias: low   - Same outcome assessed for all biomarkers - Outcome assessment independent of test - Adequate reference standard description   Concerns about applicability: low  Relevant outcomes | Risk of bias: Unclear   - All patients outcome assessed, clear exclusion criteria - Unclear if missing data - Test conduct clear on predetermined timepoint   Concerns about flow introduced bias: low | Fair |
| Santolaya, 2008 | Risk of bias: unclear  - Selection process clear  - Clear study design  - Unclear definition of fever used  - Avoided inappropriate exclusion  Concerns about applicability: low  - Appropriate study population | Risk of bias: unclear   - Unclear if index tests interpreted without knowledge of outcome - Unclear if thresholds prespecified   Concerns about applicability: low  Execution, conduct and interpretation of tests clear | Risk of bias: low   - Same outcome assessed for all biomarkers - Outcome assessment independent of test - Adequate reference standard description   Concerns about applicability: low  Relevant outcomes | Risk of bias: low   - All patients outcome assessed, clear exclusion criteria - No missing data - Test conduct clear on predetermined timepoint - Clearly described why biomarkers were excluded from in-depth analysis   Concerns about flow introduced bias: low | Fair |
| Santolaya, 2013 | Risk of bias: low  - Selection process clear  - Prospective cohort  - Clear definitions used  - Avoided inappropriate exclusion  Concerns about applicability: low  - Appropriate study population | Risk of bias: low   - Index tests interpreted without knowledge of outcome - Thresholds prespecified   Concerns about applicability: low  Execution, conduct and interpretation of tests clear | Risk of bias: low   - Same outcome assessed for all biomarkers - Outcome assessment independent of test - Adequate reference standard description   Concerns about applicability: low  Relevant outcomes | Risk of bias: unclear   - All patients outcome assessed, clear exclusion criteria - >91% received all tests at both timepoints - Test conduct clear on predetermined timepoint   Concerns about flow introduced bias: low | Good |
| Schmidt, 2007 | Risk of bias: unclear  - Selection process clear  - Prospective cohort  - Clear definitions used  - Avoided inappropriate exclusion  - Multiple episodes from same patients unclear  Concerns about applicability: low  - Appropriate study population | Risk of bias: low   - Index tests interpreted without knowledge of outcome - Thresholds prespecified   Concerns about applicability: low  Execution, conduct and interpretation of tests clear | Risk of bias: low   - Same outcome assessed for all biomarkers - Outcome assessment independent of test - Adequate reference standard description   Concerns about applicability: low  Relevant outcomes | Risk of bias: low   - All patients outcome assessed, clear exclusion criteria - Missing data clearly stated - Test conduct clear on predetermined timepoint   Concerns about flow introduced bias: low | Good |
| Secmeer, 2007 | Risk of bias: unclear   - Selection process clear - Prospective cohort - Clear definitions used, except for neutropenia - Avoided inappropriate exclusion   Concerns about applicability: low  Appropriate study population | Risk of bias: unclear   - Unclear if index tests interpreted with knowledge of outcome - Unclear if thresholds prespecified   Concerns about applicability: low   - Execution, interpretation and conduct of tests clear | Risk of bias: low   - Same outcome assessed for all biomarkers - Outcome assessment independent of test - Adequate reference standard description   Concerns about applicability: low  Relevant outcomes | Risk of bias: low   - All patients included had outcome assessed - Test conduct clear on predetermined timepoint (prior to antibiotics) - Clearly stated missing data.   Concerns about flow introduced bias: low | Fair |
| Soker, 2001 | Risk of bias: unclear  - Selection process clear  - Unclear study design, partially case-control  - Clear definitions used  - Unclear if avoided inappropriate exclusion  Concerns about applicability: low  - Appropriate study population | Risk of bias: unclear   - Unclear if index tests interpreted without knowledge of outcome - Unclear if thresholds prespecified   Concerns about applicability: low  Execution, conduct and interpretation of tests clear | Risk of bias: low   - Same outcome assessed for all biomarkers - Outcome assessment independent of test - Adequate reference standard description   Concerns about applicability: low  Relevant outcomes | Risk of bias: low   - All patients outcome assessed, clear exclusion criteria - No missing data - Test conduct clear on predetermined timepoint   Concerns about flow introduced bias: low | Fair |
| Spasova, 2005 | Risk of bias: low  - Selection process clear  - Prospective cohort  - Clear definitions used  - Avoided inappropriate exclusion  Concerns about applicability: low  - Appropriate study population | Risk of bias: unclear   - Unclear if index tests interpreted without knowledge of outcome - Thresholds prespecified   Concerns about applicability: low  Execution, conduct and interpretation of tests clear | Risk of bias: High   - Not same outcome assessed for all biomarkers - Outcome assessment independent of test - Adequate reference standard description   Concerns about applicability: low  Relevant outcomes | Risk of bias: unclear   - All patients outcome assessed, - Unclear if missing data - Test conduct not clear and not on same timepoints   Concerns about flow introduced bias: low | Poor |
| Stryjewski, 2005 | Risk of bias: low  - Selection process clear  - Unclear if prospective or retrospective cohort  - Clear definitions used  - Avoided inappropriate exclusion  Concerns about applicability: low  - Appropriate study population | Risk of bias: unclear   - Unclear if index tests interpreted without knowledge of outcome - Thresholds prespecified   Concerns about applicability: low  Execution, conduct and interpretation of tests clear | Risk of bias: low   - Same outcome assessed for all biomarkers - Outcome assessment independent of test - Adequate reference standard description   Concerns about applicability: low  Relevant outcomes | Risk of bias: low   - All patients included had outcome assessed - Test conduct clear on predetermined timepoint (prior to antibiotics) - No missing data   Concerns about flow introduced bias: low | Fair |
| Urbonas, 2012  (IL-10) | Risk of bias: unclear  - Selection process unclear  - Prospective cohort  - Clear definitions used  - Avoided inappropriate exclusion  - Multiple episodes from same patients unclear  Concerns about applicability: low  - Appropriate study population | Risk of bias: unclear   - Unclear if index tests interpreted without knowledge of outcome - Thresholds prespecified   Concerns about applicability: low  Execution, conduct and interpretation of tests clear | Risk of bias: low   - Same outcome assessed for all biomarkers - Outcome assessment independent of test - Adequate reference standard description   Concerns about applicability: low  Relevant outcomes | Risk of bias: Unclear   - All patients outcome assessed, clear exclusion criteria - Unclear if missing data - Test conduct clear on predetermined timepoint   Concerns about flow introduced bias: low | Fair |
| Urbonas, 2012  (IL-6/8) | Risk of bias: low  - Selection process clear  - Prospective cohort  - Clear definitions used  - Avoided inappropriate exclusion  Concerns about applicability: low  - Appropriate study population | Risk of bias: unclear   - Unclear if index tests interpreted with knowledge of outcome - Thresholds prespecified   Concerns about applicability: low  Execution, conduct and interpretation of tests clear | Risk of bias: low   - Same outcome assessed for all biomarkers - Outcome assessment independent of test - Adequate reference standard description   Concerns about applicability: low   - Relevant outcomes | Risk of bias: unclear   - All patients outcome assessed, - Unclear if missing data - Test conduct clear on predetermined timepoint   Concerns about flow introduced bias: low | Good |
| Urbonas, 2013 | Risk of bias: unclear  - Selection process unclear  - Prospective cohort  - Clear definitions used  - Avoided inappropriate exclusion  - Multiple episodes from same patients unclear  Concerns about applicability: low  - Appropriate study population | Risk of bias: unclear   - Unclear if index tests interpreted without knowledge of outcome - Thresholds prespecified   Concerns about applicability: low  Execution, conduct and interpretation of tests clear | Risk of bias: low   - Same outcome assessed for all biomarkers - Outcome assessment independent of test - Adequate reference standard description   Concerns about applicability: low  Relevant outcomes | Risk of bias: Unclear   - Unclear if all patients outcome assessed, - Clear exclusion criteria - Unclear if missing data - Test conduct clear on predetermined timepoint - Clear reasons for discontinuation analysis for testing of 2/4 biomarkers   Concerns about flow introduced bias: low | Fair |
| Van der Galiën, 2018 | Risk of bias: low  - Selection process clear  - Prospective cohort  - Clear definitions used  - Avoided inappropriate exclusion  Concerns about applicability: low  - Appropriate study population | Risk of bias: unclear  - Unclear if index tests interpreted without knowledge of outcome  - Thresholds prespecified  Concerns about applicability: low  - Execution, conduct and interpretation of tests clear | Risk of bias: low  - Same outcome assessed for all biomarkers  - Outcome assessment independent of test  - Adequate reference standards description  Concerns about applicability: low  - Relevant outcomes | Risk of bias: low  - All patients outcome assessed; corrected for patients excluded for inadequate blood sample sizes.  - Missing data clearly stated  - Test conduct clear on predetermined timepoints  Concerns patient flow introduced bias: low | Fair |
| Vyles, 2016 | Risk of bias: unclear  - Selection process clear  - Retrospective cohort  - Clear definitions used  - Age of paediatric patients unclear  - Clear exclusion criteria  - Avoided inappropriate exclusion  Concerns about applicability: low  - Appropriate study population | Risk of bias: high   - Index tests interpreted with knowledge of outcome - Thresholds prespecified   Concerns about applicability: low  Execution, conduct and interpretation of tests clear | Risk of bias: low   - Same outcome assessed for all biomarkers - Outcome assessment independent of test - Adequate reference standard description   Concerns about applicability: low   - Relevant outcomes | Risk of bias: high   - All patients included had outcome assessed - Missing data on cytokines, as not all were determined by standard protocol. - Test conduct clear on predetermined timepoint   Concerns about flow introduced bias: low | Poor |
| Xia, 2016 | Risk of bias: low   - Selection process clear - Retrospective cohort - Clear definitions used - Avoided inappropriate exclusion   Concerns about applicability: low  Appropriate study population | Risk of bias: high   - Index tests interpreted with knowledge of outcome - Thresholds prespecified   Concerns about applicability: low  Execution, conduct and interpretation of tests clear | Risk of bias: low   - Same outcome assessed for all biomarkers - Outcome assessment independent of test - Adequate reference standard description   Concerns about applicability: low  Relevant outcomes | Risk of bias: low   - All patients outcome assessed, clear exclusion criteria - No missing data - Test conduct clear on predetermined timepoint   Concerns about flow introduced bias: low | Fair |
| Xu, 2013 | Risk of bias: unclear  - Selection process clear  - Prospective cohort  - Clear definitions used  - Unclear exclusion criteria  - Unclear if avoided inappropriate exclusion  Concerns about applicability: low  - Appropriate study population | Risk of bias: unclear   - Unclear if index tests interpreted with knowledge of outcome - Thresholds prespecified   Concerns about applicability: low  Execution, conduct and interpretation of tests clear | Risk of bias: low   - Same outcome assessed for all biomarkers - Outcome assessment independent of test - Adequate reference standard description   Concerns about applicability: low   - Relevant outcomes | Risk of bias: low   - All patients included had outcome assessed - No missing data - Clearly state why 3 biomarkers weren’t analysed in-depth - Test conduct clear on predetermined timepoint   Concerns about flow introduced bias: low | Good |
| Xu, 2019 | Risk of bias: low   - Selection process clear - Prospective cohort - Clear definitions used - Avoided inappropriate exclusion   Concerns about applicability: low  Appropriate study population | Risk of bias: low   - Index tests interpreted without knowledge of outcome - Thresholds prespecified   Concerns about applicability: low  Execution, conduct and interpretation of tests clear | Risk of bias: low   - Same outcome assessed for all biomarkers - Outcome assessment independent of test - Adequate reference standard description   Concerns about applicability: low  Relevant outcomes | Risk of bias: low   - All patients outcome assessed, clear exclusion criteria - No missing data - Test conduct clear on predetermined timepoint   Concerns about flow introduced bias: low | Good |
